# Diagnosing Atypical Flutter in the Post-atrial Fibrillation Ablation Patient: A Case Report

**DOI:** 10.5811/cpcem.1413

**Published:** 2023-05-30

**Authors:** Alexandra Nicole Fuher, Ryan Borne, John Cunningham

**Affiliations:** * University of Colorado Anschutz Medical Campus, Department of Internal Medicine, Aurora, Colorado; † University of Colorado Health, Division of Cardiology, Colorado Springs, Colorado; ‡ Denver Health and Hospital Authority, Division of Internal Medicine, Denver, Colorado

**Keywords:** case report, atrial fibrillation, atrial flutter, typical, atypical

## Abstract

**Introduction:**

Late atrial arrhythmias after catheter ablation for atrial fibrillation occur in up to 30% of post-ablation patients and are increasingly encountered by emergency physicians. However, diagnosing the exact mechanism of the arrhythmia on the surface electrocardiogram (ECG) remains challenging due to atrial scarring leading to heterogeneous P-wave morphology.

**Case Report:**

A 74-year-old male with a history of prior catheter ablation for atrial fibrillation presented with palpitations and subacute symptoms of heart failure. The patient’s ECG revealed narrow complex tachycardia with more P waves than QRS complexes. The differential diagnosis included typical flutter, atypical flutter, and focal atrial tachycardias with 2:1 conduction block. P waves were positive in V1 and across all precordial leads (absent precordial transition). This favors atypical flutter originating from the left atrium over typical cavotricuspid isthmus-dependent right atrial flutter. Transthoracic echocardiogram showed a reduced ejection fraction due to tachycardia-mediated cardiomyopathy. The patient underwent a repeat electrophysiology study and ablation, which confirmed the presence of an atypical flutter circuit using the mitral annulus, known as perimitral flutter. Repeat catheter ablation resulted in maintenance of sinus rhythm. At follow-up, his ejection fraction recovered.

**Conclusion:**

Recognizing ECG findings suggestive of atypical flutter impacts initial emergency department decisions and triage as atypical flutter post-atrial fibrillation ablation is frequently resistant to rate-controlling medications and often requires cardiology and/or electrophysiology consultation if available.

## INTRODUCTION

Catheter ablation with pulmonary vein isolation is increasingly used in the management of paroxysmal atrial fibrillation (AF). Post-ablation late atrial tachycardias are common, occurring in up to 30% of patients.^1^,^2^ As a result, emergency physicians and other acute care clinicians are encountering post-ablation arrhythmias more frequently. The electrocardiogram (ECG) interpretation in this population, however, is challenging. Analysis of P-wave morphology to determine the mechanism of arrhythmia is limited by the altered atrial conduction, which results from atrial scarring. This case report demonstrates ECG findings that distinguish atypical flutter circuits originating from the left atrium (LA) from typical atrial flutter in patients who are post ablation.

## CASE REPORT

A 74-year-old male with a history of symptomatic drug-refractory paroxysmal AF treated with a catheter ablation with pulmonary vein isolation 14 years prior and a second catheter ablation two years prior, presented to the emergency department (ED) with three months of progressive shortness of breath and palpitations. Upon presentation, he was afebrile, his heart rate was 137 beats per minute, respiratory rate was 18 breaths per minute, blood pressure was 106/76 millimeters of mercury, and oxygen saturation was 90% on room air. Cardiovascular examination revealed tachycardia with normal heart sounds and no murmurs, rubs or gallops. Jugular venous pressure was elevated. Pulmonary exam was negative for rales, and there was no lower extremity edema.

**Image 1:**
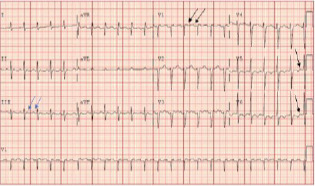
Electrocardiogram on presentation. Two P waves occur for every QRS complex. Upright flutter waves in the precordial leads remain positive across all precordial leads (noted by black arrows). Upright inferior P waves in the inferior leads, annotated with blue arrows.

The ECG (Image 1) revealed narrow complex tachycardia with more P waves than QRS complexes. The differential diagnosis included typical and atypical atrial flutter and focal atrial tachycardias (AT) with 2:1 conduction block. Notably, P waves are positive in V1 and across all precordial leads (absent precordial transition), favoring atypical flutter from the left atrium over typical cavotricuspid isthmus (CTI)-dependent right atrial flutter. Positive flutter waves in the inferior leads indicate high to low atrial activation, most often caused by counterclockwise perimitral flutter and left atrial (LA) roof-dependent AT. Focal AT, due to enhanced automaticity from an ectopic focus, cannot be ruled out based on the ECG findings; however, reentrant arrhythmias are more common post-AF ablation.

*CPC-EM Capsule*

What do we already know about this clinical entity?

*Late atrial arrhythmias after atrial fibrillation ablation occur in up to 30% of post-ablation patients. However, the diagnosis remains challenging due to heterogeneous P wave morphology.*

What makes this presentation of disease reportable?

*This case highlights the electrocardiogram (ECG) findings suggestive of atypical flutter in a patient with prior catheter ablation.*

What is the major learning point?

*The absence of precordial transition favors atypical flutter originating in the left atrium in the post-ablation patient, among other ECG findings.*

How might this improve emergency medicine practice?

*Atypical flutter is often resistant to control medications. Symptomatic patients presenting with atypical flutter warrant early consultation or referral to cardiac electrophysiology.*

**Image 2:**
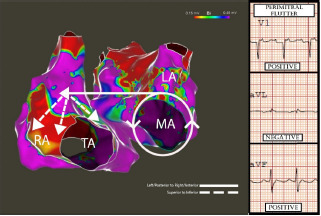
The patient’s intracardiac voltage mapping is adjacent to the associated P-wave polarity of electrocardiogram leads V1, aVF, and aVL. Solid white lines with arrows indicate the counterclockwise perimitral annulus flutter circuit. Solid lines indicate left to right and posterior to anterior initial atrial activation toward the right atria resulting in a positive P wave in lead V1 and negative P wave in lead aVL. Dashed lines indicate superior to inferior RA depolarization resulting in positive inferior P waves. *MA*, mitral annulus; *TA*, tricuspid annulus; *RA*, right atrium; *LA*, left atrium.

A transthoracic echocardiogram (TTE) revealed a severely reduced ejection fraction of 20% without valvular disease or wall motion abnormalities. The patient was started on amiodarone and underwent direct current cardioversion. An electrophysiology study revealed perimitral isthmus flutter. Image 2 indicates how the pattern of right atrial activation correlates with the ECG flutter wave morphology on the patient’s initial ECG. Repeat catheter ablation successfully terminated the arrhythmia. One month later, a follow-up TTE showed a recovered ejection fraction of 50%, indicating a likely tachycardia-mediated cardiomyopathy.

## DISCUSSION

Delayed atrial arrhythmias post-AF ablation present a challenge as the degree of atrial scarring and location of prior radiofrequency ablation sites result in variable P-wave morphology. Most post-ablation arrhythmias are caused by either macroreentry or localized microreentrant atrial tachycardias (circuits <3 centimeters in diameter), and true focal AT due to an ectopic focus is less common.^3^ The most common macroreentrant circuits post-ablation include typical atrial flutter, perimitral flutter, and left atrial-roof dependent reentry.^3^ Reentry results from either gaps in ablation lines or ablation lesions that act as obstacles resulting in tunneled conduction through one or more isthmuses within the left atrium, thereby allowing reentry.^4^ Despite limitations, the surface ECG is essential when initially determining the type of arrhythmia post catheter ablation. The ECG can frequently differentiate typical from atypical atrial flutter originating from the left atrium. This is important as it impacts clinical management as the outcomes of repeat ablation differ between typical and atypical flutter.^3^,^5^

**Image 3:**
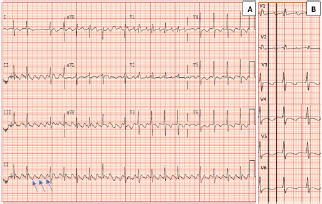
**A)** Electrocardiogram demonstrating typical cavotricuspid-isthmus dependent flutter in a patient with a prior ablation. Blue arrows indicate negatively directed inferior flutter waves. **B)** Demonstrates precordial transition from positive flutter waves to negative within the solid, vertical black lines. Typically, the negative flutter waves in the left sided leads proceeds the positive flutter wave in V1.^3^

Typical atrial flutter is defined as a macroreentrant circuit (most often counterclockwise) around the tricuspid annulus, using the CTI.^5^ This results in negative flutter waves in the inferior leads and initial positive flutter waves in the precordial leads that transition to negative by V6 (Image 3).^3^ The classic “sawtooth” pattern with negative flutter waves in the inferior leads may be absent in over half of patients with CTI-dependent flutter post-ablation. However, precordial transition is 98% specific for typical counterclockwise flutter with a high negative predictive value of 95%.^3^

Atypical atrial flutter refers to macroreentrant tachycardias that are not CTI-dependent. Atypical flutter circuits can originate in the left or right atrium, usually around an atrial scar in patients with structural heart disease.^5^ Multiple circuits may be present in the same patient. Studies suggest that P-wave morphology in post-PVI arrhythmias primarily results from right atrial activation, likely due to an electrically inert LA scar with loss of electrical forces toward the posterior LA wall.^6^ Acknowledging the relationship between the left and right atria is also posterior to anterior explains the positive P waves in V1 in LA flutter (Image 1). Negative P waves in any precordial lead suggests a right atrial circuit over a left atrial circuit, with 83% and 100% sensitivity and specificity, respectively.^1^,^4^ Therefore, the absence of precordial transition or negative precordial flutter waves in Image 1 suggests the left atrial origin of the arrhythmia.

However, an electrophysiology study is needed to map the exact reentrant circuit. Flutter wave polarity in the inferior leads does not separate typical from atypical flutter but rather indicates that the macroreentrant circuit initially activated the superior right atrium with subsequent superior to inferior right atrial activation.^6^ P-wave polarity in leads I and aVL are unreliable when distinguishing post-ablation macroreentrant tachycardias, likely due to the degree of left atrial scarring.^3^ Surface ECG cannot reliably separate LA roof-dependent reentry from PMFL, highlighting the need for electrophysiology referral.

A notable feature in Image 1 is the lack of a typical undulating flutter wave present in Image 3. A discrete isoelectric interval greater than 80 milliseconds in all leads favors focal AT over macroreentrant rhythms. However, nearly a quarter of post-ablation macroreentrant arrhythmias will have discrete isoelectric intervals in all leads.^3^ Atrial scarring may result in decreased flutter-wave voltages leading to ECG findings more characteristic of AT. Thus, an electrophysiology study with intracardiac mapping is needed for definitive diagnosis when managing post-ablation arrhythmias.^3^,^5^

Distinguishing LA atypical flutter from an arrhythmia originating in the right atrium impacts clinical management as atypical flutter is often resistant to rate-controlling and antiarrhythmic medications and frequently requires repeat catheter ablation.^3^,^7^ Therefore, earlier consultation with cardiology is often required to manage these patients. Ablation of a left atrial atypical flutter post-pulmonary vein isolation has a lower acute success rate and higher major complication rate than a typical flutter ablation due to the increased complexity of the procedure and the need for septal puncture to perform electroanatomic mapping and ablation within the left atrium.^5^

## CONCLUSION

Late post-ablation arrhythmias are increasingly common as the number of catheter ablation procedures performed for AF increases. The presence of upright P waves across all precordial leads favors atypical flutter from the left atrium over typical CTI dependent right atrial flutter. Once recognized, atypical flutter, including perimitral flutter, is often resistant to rate and rhythm controlling medications. Patients with atrial fibrillation or typical flutter who are asymptomatic once rate controlled or who spontaneously convert in the emergency department are often appropriate for outpatient follow-up with primary care, with cardiology follow-up based on symptoms and clinical course. Symptomatic patients presenting to the ED with atypical flutter warrant early consultation or referral to cardiac electrophysiology for definitive diagnosis and management.
